# Does institutional delivery help in improving infant and child health care practices and health promotion related parameters? A study from Bellary, Karnataka

**DOI:** 10.1186/1753-6561-6-S5-O22

**Published:** 2012-09-28

**Authors:** Animesh Jain, B Shantharam Baliga, Suchetha Rao, M Veera Shankar, BK Srikanth

**Affiliations:** 1 Department of Community Medicine, Kasturba Medical College, Mangalore, India; 2 Department of Paediatrics, Kasturba Medical College, Mangalore, India; 3 Department of Paediatrics, Vijayanagara Institute of Medical Sciences, Bellary, India

## Introduction

In spite of emphasis on institutional delivery, a considerable number of births are being conducted at home and by traditional birth attendant (TBA). Institutional delivery is hypothesised to improve health seeking behaviour and health care practices related to infant and child care. In this paper we report that in a poorly performing district institutional delivery does not seem to change the health care practices of the people.

## Methods

The data presented here are a part of a larger two year long study at Bellary and Dakshina Kannada districts of Karnataka. The results reported pertain to the cross-sectional community-based house-to-house survey carried out among 2,158 households in Bellary regarding births during the five-year period before survey among ever-married women. The study has obtained ethics approval.

## Results

A total of 1,010 deliveries were at an institution, while 1,148 deliveries took place at home. Of these, 891 deliveries were conducted by TBAs. Majority of mothers who delivered with the help of a TBA had not been explained about breastfeeding and its importance.

Delayed breastfeeding beyond two hours after birth was seen in 38% of institutional deliveries as opposed to 62% home deliveries. Though 79% babies delivered at home were given pre-lacteal feeds, a large number (57%) of those delivered at institution also received pre-lacteal feeds. The weaning practices were also very similar in both the groups. Majority of the women delivered at home (by TBAs) had undergone antenatal check up by either a doctor (private 41% and government 21%) or a nurse (12.7%). More than half of the houses where delivery was at home or by a TBA were in close proximity (within 5 km in 62 %) to a health facility.

Treatment-seeking behaviour for childhood illness was similar in the institutional and the home birth group. The majority went to private clinic or hospitals (55% home delivery vs. 60% hospital) followed by chemist shop (without prescription) and alternate system of medicine.

## Discussion

Health seeking behaviour and health care practices depend on the beliefs and awareness among people. Availing antenatal care is hypothesised to promote institutional delivery but this did not seem to be the case in Bellary. Institutional deliveries too did not help in doing away with practices related to delayed breastfeeding and supplementary feeding as well as pre-lacteal feeds. Mere emphasis on institutional delivery seemed to be ineffective in changing the perception and beliefs of people leading to practices, which may be harmful to child’s health. Non-utilization of health facilities for delivery in spite of proximity calls for a relook at the availability, affordability and quality of services at these facilities. Institutional delivery *per se* may not be the answer for infant and child care practices. Sustained efforts by means of campaigns and awareness activities coupled with the use of mass media may help in improving the health care practices. Health systems research and study of local traditions are needed before implementing a tailor-made strategy for improving the health care practices and behaviour in a district/region.

## Competing interests

Authors declare that they have no conflict of interest.

## Funding statement

This study was funded by the Division of Health System Research of the Indian Council of Medical Research, New Delhi, India.

